# Supplementary material for: Complementary Feeding Practices: Recommendations of Pediatricians for Infants with and without Allergy Risk
Source: Nutrients. 2024 Jan 12;16(2):239. doi: 10.3390/nu16020239 (PMC10819658; doi:10.3390/nu16020239)
Supplement: Supplementary file 1 [file nutrients-16-00239-s001.zip › nutrients-2774786-SI.pdf]

Supplement

Supplementary Table S1

Demographic characteristics of the study participants

	**Total (n=233)**
Sex	
Male	56 (24%)
Female	177 (76%)
Experience	
Low	112 (48%)
High	121 (52%)
Years of experience	15 (6.5-24)
Subspecialty	
No	174 (75%)
Yes	59 (25%)
Subspecialty	
No	173 (74%)
Neonatology	22 (9%)
Allergology	8 (3%)
Pulmonology	8 (3%)
Infectious disease	2 (1%)
Intensive care	6 (3%)
Developmental	3 (1.5%)
Gastroenterology	1 (0.5%)
Puberty	2 (1%)
Hematology	3 (1.5%)
Immunology	1 (0.5%)
Homeopathy	1 (0.5%)
Endocrinology	1 (0.5%)
Breastfeeding	3 (1.5%)
Location	
Rural	129 (55%)
Urban	104 (45%)

Place of work<br>Abroad<br>Attica<br>Macedonia<br>Epirus<br>Central Greece<br>Island<br>The Peloponnese<br>Thrace<br>Cyprus<br>Ped. Hospital<br>Private Practice<br>University<br>n/a	 5 (2%)<br>65 (28%)<br>38 (16%)<br>6 (3%)<br>15 (7%)<br>17 (7%)<br>7 (3%)<br>2 (1%)<br>3 (1.5%)<br>29 (12%)<br>37 (16%)<br>1 (0.5%)<br>8 (3%)
Parentship<br>No<br>Yes	 33 (14%)<br>200 (86%)
No of Children	2 (1-2)
Healthy-children waiting period Low<br>High	 126 (54%)<br>107 (46%)
Healthy-children waiting period duration	2 (2-3)
Allergic-children waiting period<br>Low<br>High	 24 (10%)<br>209 (90%)
Allergic-children waiting period duration	5 (3-6)

Supplementary Table S2 Age of Introduction of Solid Food as Recommended by the Pediatricians

	Total (N=233)			
VitD	0 (0-0)		Potato	6 (5-6)
Probiotics	0 (0-2)		Corn	6 (6-8)
N3	12 (7.5-18)		Peas	6 (6-7)
Multivitamins	18 (12-18)		White rice	6 (5-6)
VitA	18 (12-18)		Whole-grain rice	6 (5-7)
VitC	18 (12-18)		Oat	6 (6-7)
Iron	4 (0-6)		Whole-wheat products	7 (6-8)
Olive oil	6 (5-6)		White-wheat products	7 (6-7)
Butter	12 (10-15)		Trahanas frumenty	7 (6-8)
Olives	12 (10-12)		Baby biscuits without sugar	8 (7-12)
Seed oils	12 (12-18)		Baby biscuits with sugar	13 (12-18)
Margarine	12 (12-18)		Gluten-free oat	6 (5-7)
Almonds	9 (8-12)		Carrot	6 (5-6)
Walnuts	9 (8-12)		Zucchini	6 (5-6)
Sunflower/pumpkin seeds	10 (8-14)		Green leafy vegetables	6 (5-6)
Pistachios	10 (8-15)		Cabbage	6 (5-6)
Peanuts	10 (8-15)		Cauliflower	6 (5-6)
Hazelnuts	10 (8-12)		Tomato	6 (5-7)
Cashew nuts	10 (8-15)		Beetroot	6 (5-7)
Sesame	10 (8-12)		Spinach	6 (5-7)
Almond butter	9 (7-12)		Pepper	6 (5-7)
Hazelnut butter	9 (7-12)		Eggplant	6 (5-7)
Peanut butter	9 (7-12)		Pear	5 (5-6)
Tahini	10 (8-12)		Apple	5 (5-6)
Cod	9 (8-11)		Banana	5 (5-6)
Anchovy	10 (8-12)		Orange	6 (5-7)
Dover sole	9 (7-10)		Kiwi	6 (6-8)
Sardine	10 (8-12)		Pomegranate	7 (6-8)
Sea bass	9 (8-11)		Mandarin	6 (6-7)
Hake	9 (7-10)		Apricot	6 (5-6)
Calamari	12 (10-13)		Cherry	6 (6-7)
Salmon	11 (8-12)		Peach/nectarine	6 (5-7)
Octopus	12 (10-14)		Raisins	9 (6-12)
Shrimps	12 (9-13)		Grapes	6 (6-8)
Mussels	12 (10-18)		Strawberry	8 (6-12)
Canned tuna	13 (12-18)		Fresh orange juice	8 (6-10)
Beans	8 (7-11)		Fresh berries	7 (6-10)
Lentils	8 (7-10)		Fig	7 (6-11)
Chickpeas	8 (7-10)		Fresh mixed fruit juice	10 (7-12)
Fava	8 (7-11)		Watermelon	6 (6-7)
Hard-boiled egg	8 (7-11)		Melon	6 (6-7)
Omelet or egg eye	11 (9-12)		Dried berries	10 (7-12)
Raw egg	13 (11-18)		Dried apricots	9 (6-12)
Egg-lemon sauce	12 (9-12)		Dried dates	8 (6-12)
Cow milk	12 (12-13)		Dried figs	10 (7-12)
Goat milk	12 (12-13)		Boiled food	6 (5-6)
Cow-milk yogurt	8 (7-12)		Family food without salt	12 (9-12)
Traditional yogurt	8 (7-12)		Family food with salt	13 (12-18)
Sheep-milk yogurt	8 (7-12)		Baby-led weaning	8 (6-12)
Drained yogurt	9 (7-12)		Mashed food	6 (5-6)
Fruit yogurt	9 (7-12)		Fork-mashed food	9 (8-10)

Kids' yogurt	7 (6-8)		Food in pieces	10 (9-12)
Cottage cheese	11 (8-12)		Roasted food	12 (9-12)
Cream cheese	12 (8-12)		Family food with salt	13 (12-18)
Manouri cheese	9 (7-12)		Fried food	12 (12-18)
Katiki cheese	12 (8-12)		Sugar sweets	15 (12-18)
Feta cheese	12 (10-12)		Honey	12 (12-13)
Kaseri/graviera/kefalotyri cheese	12 (10-13)		Salt	12 (12-18)
Gouda cheese	12 (9-12)		Spices	12 (8-13)
Sour cream	13 (12-18)		Ready meals in jar	12 (8-13)
Ariani	12 (10-13)			
Kefir	12 (8-12)			
Chicken	6 (6-6)			
Rabbit	7 (6-8)			
Lamb	7 (6-8)			
Beef	6 (6-6)			
Pork	10 (7-12)			
Hare or wild boar	9 (7-12)			
Birds	9 (7-12)			
Liver	9 (7-12)			
Cold cuts/salami/ham	18 (12-18)			

Supplementary Table S3: Differences based on location of practice

	**Semi-urban or rural (N = 129)**	**Urban (N = 104)**	**p-value**
Rabbit	6 (6-8)	7 (6-8)	0.02
Kaseri/graviera/kefalotyri cheese	12 (11-14)	12 (10-12)	0.04
Ariani	12 (9-12)	12 (12-14)	0.04
Hazelnuts	9 (7-12)	12 (9-13)	0.03
Hazelnut butter	9 (7-11)	10 (8-12)	0.04
Iron supplement	3 (0-6)	4 (4-6)	0.04

Supplementary Table S4: Differences based on parentship

**Food**	**Total (N=233)**	**No (N=33)**	**Yes (N=200)**	**p**
Cherry	6 (6-7)	6 (6-7)	6 (6-7)	0.04
Fresh orange juice	8 (6-10)	8 (6-10)	7 (6-11)	0.02
Whole-wheat products	7 (6-8)	7 (6-8)	7 (6-7)	0.008
White-wheat products	7 (6-7)	7 (6-7)	7 (6-7)	0.02
Cottage cheese	11 (8-12)	10 (8-12)	12 (8-12)	0.023
Mussels	12 (10-18)	12 (10-18)	12 (10-15)	0.04
Baby-led weaning	8 (6-12)	7 (6-12)	8 (6-12)	0.003
Fork-mashed food	9 (8-10)	9 (8-10)	9 (8-10)	0.04

Supplementary Table S5

DEPENDENT VARIABLE: ALMONDS/ALMOND BUTTER

INDEPENDENT VARIABLE: SEX, EXPERIENCE, SUBSPECIALTY, LOCATION, PARENTSHIP

Variables in the Equation

		B	S.E.	Wald	df	Sig.	Exp(B)	95% C.I. for EXP(B)	
								Lower	Upper
Step 1[a]	SEX(1)	.721	.516	1.952	1	.162	2.056	.748	5.654
	EXPERIENCE(1)	-1.471	.503	8.558	1	.003	.230	.086	.616
	SUBSPECIALTY (1)	-1.505	.399	14.191	1	.000	.222	.102	.486
	LOCATION(1)	-.836	.407	4.210	1	.040	.434	.195	.963
	PARENTSHIP(1)	.848	.678	1.563	1	.211	2.334	.618	8.812

a. Variable(s) entered on step 1: SEX, EXPERIENCE, SUBSPECIALTY, LOCATION, PARENTSHIP.

DEPENDENT VARIABLE: WALNUTS

INDEPENDENT VARIABLE: SEX, EXPERIENCE, SUBSPECIALTY, LOCATION, PARENTSHIP

Variables in the Equation

		B	S.E.	Wald	df	Sig.	Exp(B)	95% C.I. for EXP(B)	
								Lower	Upper
Step 1[a]	SEX(1)	.696	.461	2.277	1	.131	2.006	.812	4.954
	EXPERIENCE(1)	-.649	.400	2.624	1	.105	.523	.238	1.146
	SUBSPECIALTY (1)	-.901	.353	6.519	1	.011	.406	.203	.811
	LOCATION(1)	-.735	.367	4.016	1	.045	.480	.234	.984
	PARENTSHIP(1)	.372	.602	.382	1	.537	1.451	.446	4.724

a. Variable(s) entered on step 1: SEX, EXPERIENCE, SUBSPECIALTY, LOCATION, PARENTSHIP.

DEPENDENT VARIABLE: PISTACHIOS

INDEPENDENT VARIABLE: SEX, EXPERIENCE, SUBSPECIALTY, LOCATION, PARENTSHIP

Variables in the Equation

		B	S.E.	Wald	df	Sig.	Exp(B)	95% C.I. for EXP(B) Lower	95% C.I. for EXP(B) Upper
Step 1[a]	SEX(1)	-.010	.519	.000	1	.985	.990	.358	2.736
	EXPERIENCE(1)	-.867	.413	4.406	1	.036	.420	.187	.944
	SUBSPECIALTY(1)	-.353	.379	.869	1	.351	.703	.334	1.476
	LOCATION(1)	-.402	.372	1.165	1	.280	.669	.322	1.388
	PARENTSHIP(1)	.231	.663	.122	1	.727	1.260	.344	4.621

a. Variable(s) entered on step 1: SEX, EXPERIENCE, SUBSPECIALTY, LOCATION, PARENTSHIP.

DEPENDENT VARIABLE: PEANUTS/PEANUT BUTTER

INDEPENDENT VARIABLE: SEX, EXPERIENCE, SUBSPECIALTY, LOCATION, PARENTSHIP

Variables in the Equation

		B	S.E.	Wald	df	Sig.	Exp(B)	95% C.I. for EXP(B) Lower	95% C.I. for EXP(B) Upper
Step 1[a]	SEX(1)	-.503	.522	.928	1	.335	.605	.217	1.683
	EXPERIENCE(1)	-1.521	.450	11.412	1	.001	.218	.090	.528
	SUBSPECIALTY(1)	-.339	.365	.866	1	.352	.712	.349	1.456
	LOCATION(1)	-.716	.367	3.799	1	.051	.489	.238	1.004
	PARENTSHIP(1)	.245	.632	.150	1	.699	1.277	.370	4.407

a. Variable(s) entered on step 1: SEX, EXPERIENCE, SUBSPECIALTY, LOCATION, PARENTSHIP.

DEPENDENT VARIABLE: SESAME/TAHINI

INDEPENDENT VARIABLE: SEX, EXPERIENCE, SUBSPECIALTY, LOCATION, PARENTSHIP

Variables in the Equation

		B	S.E.	Wald	df	Sig.	Exp(B)	95% C.I. for EXP(B)	
								Lower	Upper
Step 1[a]	SEX(1)	-1.002	.594	2.847	1	.092	.367	.115	1.176
	EXPERIENCE(1)	-1.861	.494	14.204	1	.000	.156	.059	.409
	SUBSPECIALTY (1)	-.359	.377	.907	1	.341	.698	.334	1.462
	LOCATION(1)	-.765	.380	4.062	1	.044	.465	.221	.979
	PARENTSHIP(1)	.197	.703	.079	1	.779	1.218	.307	4.826

a. Variable(s) entered on step 1: SEX, EXPERIENCE, SUBSPECIALTY, LOCATION, PARENTSHIP.

DEPENDENT VARIABLE: HAZELNUT BUTTER

INDEPENDENT VARIABLE: SEX, EXPERIENCE, SUBSPECIALTY, LOCATION, PARENTSHIP

Variables in the Equation

		B	S.E.	Wald	df	Sig.	Exp(B)	95% C.I. for EXP(B)	
								Lower	Upper
Step 1[a]	SEX(1)	-1.473	.803	3.364	1	.067	.229	.047	1.106
	EXPERIENCE(1)	-1.158	.533	4.715	1	.030	.314	.110	.893
	SUBSPECIALTY(1)	-.524	.473	1.228	1	.268	.592	.234	1.496
	LOCATION(1)	-1.105	.482	5.260	1	.022	.331	.129	.852
	PARENTSHIP(1)	.842	.761	1.224	1	.269	2.321	.522	10.314

a. Variable(s) entered on step 1: SEX, EXPERIENCE, SUBSPECIALTY, LOCATION, PARENTSHIP.

DEPENDENT VARIABLE: SEAFOOD

INDEPENDENT VARIABLE: SEX, EXPERIENCE, SUBSPECIALTY, LOCATION, PARENTSHIP

Variables in the Equation

		B	S.E.	Wald	df	Sig.	Exp(B)	95% C.I. for EXP(B)	
								Lower	Upper
Step 1[a]	SEX(1)	-18.234	4990.962	.000	1	.997	.000	.000	.
	EXPERIENCE(1)	-18.360	3530.094	.000	1	.996	.000	.000	.
	SUBSPECIALTY (1)	-2.305	.746	9.553	1	.002	.100	.023	.430
	LOCATION(1)	-2.705	1.037	6.801	1	.009	.067	.009	.511
	PARENTSHIP(1)	-15.098	6209.920	.000	1	.998	.000	.000	.

a. Variable(s) entered on step 1: SEX, EXPERIENCE, SUBSPECIALTY, LOCATION, PARENTSHIP.

DEPENDENT VARIABLE: CALAMARI

INDEPENDENT VARIABLE: SEX, EXPERIENCE, SUBSPECIALTY, LOCATION, PARENTSHIP

Variables in the Equation

		B	S.E.	Wald	df	Sig.	Exp(B)	95% C.I. for EXP(B)	
								Lower	Upper
Step 1[a]	SEX(1)	-.286	.542	.279	1	.598	.751	.259	2.174
	EXPERIENCE(1)	-1.686	.428	15.509	1	.000	.185	.080	.429
	SUBSPECIALTY(1)	.239	.389	.376	1	.540	1.270	.592	2.722
	LOCATION(1)	-.268	.368	.529	1	.467	.765	.372	1.574
	PARENTSHIP(1)	.065	.618	.011	1	.917	1.067	.318	3.581

a. Variable(s) entered on step 1: SEX, EXPERIENCE, SUBSPECIALTY, LOCATION, PARENTSHIP.

DEPENDENT VARIABLE: BEANS

INDEPENDENT VARIABLE: SEX, EXPERIENCE, SUBSPECIALTY, LOCATION, PARENTSHIP

Variables in the Equation

		B	S.E.	Wald	df	Sig.	Exp(B)	95% C.I. for EXP(B)	
								Lower	Upper

Step 1[a]	SEX(1)	-.951	.793	1.440	1	.230	.386	.082	1.826
	EXPERIENCE(1)	-1.756	.669	6.900	1	.009	.173	.047	.640
	SUBSPECIALTY(1)	-1.446	.464	9.715	1	.002	.236	.095	.585
	LOCATION(1)	-1.814	.567	10.247	1	.001	.163	.054	.495
	PARENTSHIP(1)	.110	1.134	.009	1	.923	1.116	.121	10.303

a. Variable(s) entered on step 1: SEX, EXPERIENCE, SUBSPECIALTY, LOCATION, PARENTSHIP.

DEPENDENT VARIABLE: LENTILS

INDEPENDENT VARIABLE: SEX, EXPERIENCE, SUBSPECIALTY, LOCATION, PARENTSHIP

Variables in the Equation

								95% C.I. for EXP(B)	
		B	S.E.	Wald	df	Sig.	Exp(B)	Lower	Upper
Step 1[a]	SEX(1)	-.784	1.164	.453	1	.501	.457	.047	4.473
	EXPERIENCE(1)	-18.582	3657.451	.000	1	.996	.000	.000	.
	SUBSPECIALTY (1)	-2.440	.762	10.253	1	.001	.087	.020	.388
	LOCATION(1)	-2.646	1.043	6.432	1	.011	.071	.009	.548
	PARENTSHIP(1)	-15.796	6377.711	.000	1	.998	.000	.000	.

a. Variable(s) entered on step 1: SEX, EXPERIENCE, SUBSPECIALTY, LOCATION, PARENTSHIP.

DEPENDENT VARIABLE: HARD-BOILED EGG

INDEPENDENT VARIABLE: SEX, EXPERIENCE, SUBSPECIALTY, LOCATION, PARENTSHIP

Variables in the Equation

								95% C.I. for EXP(B)	
		B	S.E.	Wald	df	Sig.	Exp(B)	Lower	Upper
Step 1[a]	SEX(1)	.077	.364	.045	1	.832	1.081	.529	2.207

EXPERIENCE(1)	-.622	.311	4.011	1	.045	.537	.292	.987
SUBSPECIALTY (1)	-.576	.269	4.588	1	.032	.562	.332	.952
LOCATION(1)	-.445	.281	2.502	1	.114	.641	.369	1.112
PARENTSHIP(1)	-.629	.584	1.159	1	.282	.533	.170	1.675

a. Variable(s) entered on step 1: SEX, EXPERIENCE, SUBSPECIALTY, LOCATION, PARENTSHIP.

DEPENDENT VARIABLE: OMELETTE OR EGG EYE

INDEPENDENT VARIABLE: SEX, EXPERIENCE, SUBSPECIALTY, LOCATION, PARENTSHIP

Variables in the Equation

		B	S.E.	Wald	df	Sig.	Exp(B)	95% C.I. for EXP(B) Lower	Upper
Step 1[a]	SEX(1)	.662	.430	2.367	1	.124	1.939	.834	4.506
	EXPERIENCE(1)	-.455	.324	1.969	1	.161	.634	.336	1.198
	SUBSPECIALTY (1)	.912	.321	8.104	1	.004	2.490	1.329	4.668
	LOCATION(1)	-.235	.312	.571	1	.450	.790	.429	1.455
	PARENTSHIP(1)	.673	.576	1.365	1	.243	1.960	.634	6.062

a. Variable(s) entered on step 1: SEX, EXPERIENCE, SUBSPECIALTY, LOCATION, PARENTSHIP.

DEPENDENT VARIABLE: COW-MILK YOGURT

INDEPENDENT VARIABLE: SEX, EXPERIENCE, SUBSPECIALTY, LOCATION, PARENTSHIP

Variables in the Equation

		B	S.E.	Wald	df	Sig.	Exp(B)	95% C.I. for EXP(B) Lower	Upper
Step 1[a]	SEX(1)	.093	.441	.044	1	.833	1.097	.462	2.606

	EXPERIENCE(1)	-.843	.363	5.398	1	.020	.431	.211	.876
	SUBSPECIALTY (1)	-.530	.333	2.528	1	.112	.589	.306	1.131
	LOCATION(1)	.218	.337	.417	1	.518	1.243	.642	2.407
	PARENTSHIP(1)	-.320	.576	.308	1	.579	.726	.235	2.246

a. Variable(s) entered on step 1: SEX, EXPERIENCE, SUBSPECIALTY, LOCATION, PARENTSHIP.

DEPENDENT VARIABLE: COW MILK

INDEPENDENT VARIABLE: SEX, EXPERIENCE, SUBSPECIALTY, LOCATION, PARENTSHIP

Variables in the Equation

								95% C.I. for EXP(B)	
		B	S.E.	Wald	df	Sig.	Exp(B)	Lower	Upper
Step 1[a]	SEX(1)	-.391	.375	1.090	1	.296	.676	.324	1.409
	EXPERIENCE(1)	-.458	.308	2.205	1	.138	.633	.346	1.158
	SUBSPECIALTY (1)	-.165	.288	.329	1	.566	.848	.482	1.491
	LOCATION(1)	-.055	.294	.035	1	.852	.946	.531	1.686
	PARENTSHIP(1)	-1.337	.656	4.157	1	.041	.263	.073	.950

a. Variable(s) entered on step 1: SEX, EXPERIENCE, SUBSPECIALTY, LOCATION, PARENTSHIP.

DEPENDENT VARIABLE: CREAM CHEESE

INDEPENDENT VARIABLE: SEX, EXPERIENCE, SUBSPECIALTY, LOCATION, PARENTSHIP

Variables in the Equation

								95% C.I. for EXP(B)	
		B	S.E.	Wald	df	Sig.	Exp(B)	Lower	Upper
Step 1[a]	SEX(1)	-.444	.497	.799	1	.371	.642	.242	1.698
	EXPERIENCE(1)	.140	.417	.113	1	.737	1.151	.508	2.605

		B	S.E.	Wald	df	Sig.	Exp(B)	Lower	Upper
	SUBSPECIALTY (1)	.828	.379	4.756	1	.029	2.288	1.087	4.812
	LOCATION(1)	.495	.370	1.795	1	.180	1.641	.795	3.387
	PARENTSHIP(1)	.047	.652	.005	1	.942	1.049	.292	3.763

a. Variable(s) entered on step 1: SEX, EXPERIENCE, SUBSPECIALTY, LOCATION, PARENTSHIP.

DEPENDENT VARIABLE: GOUDA CHEESE

INDEPENDENT VARIABLE: SEX, EXPERIENCE, SUBSPECIALTY, LOCATION, PARENTSHIP

Variables in the Equation

								95% C.I. for EXP(B)	
		B	S.E.	Wald	df	Sig.	Exp(B)	Lower	Upper
Step 1[a]	SEX(1)	-1.423	.636	5.010	1	.025	.241	.069	.838
	EXPERIENCE(1)	-.316	.373	.715	1	.398	.729	.351	1.516
	SUBSPECIALTY(1)	-.695	.335	4.297	1	.038	.499	.259	.963
	LOCATION(1)	-.478	.343	1.937	1	.164	.620	.316	1.215
	PARENTSHIP(1)	-.372	.687	.293	1	.589	.690	.179	2.650

a. Variable(s) entered on step 1: SEX, EXPERIENCE, SUBSPECIALTY, LOCATION, PARENTSHIP.

DEPENDENT VARIABLE: WHEAT PRODUCTS

INDEPENDENT VARIABLE: SEX, EXPERIENCE, SUBSPECIALTY, LOCATION, PARENTSHIP

Variables in the Equation

								95% C.I. for EXP(B)	
		B	S.E.	Wald	df	Sig.	Exp(B)	Lower	Upper
Step 1[a]	SEX(1)	-1.893	.762	6.175	1	.013	.151	.034	.670
	EXPERIENCE(1)	-1.027	.381	7.279	1	.007	.358	.170	.755
	SUBSPECIALTY(1)	-.626	.321	3.811	1	.051	.535	.285	1.003
	LOCATION(1)	-.648	.339	3.644	1	.056	.523	.269	1.017
	PARENTSHIP(1)	1.186	.537	4.884	1	.027	3.274	1.144	9.373

a. Variable(s) entered on step 1: SEX, EXPERIENCE, SUBSPECIALTY, LOCATION, PARENTSHIP.

DEPENDENT VARIABLE: TRAHANAS

INDEPENDENT VARIABLE: SEX, EXPERIENCE, SUBSPECIALTY, LOCATION, PARENTSHIP

Variables in the Equation

		B	S.E.	Wald	df	Sig.	Exp(B)	95% C.I. for EXP(B)	
								Lower	Upper
Step 1[a]	SEX(1)	-.494	.374	1.741	1	.187	.610	.293	1.271
	EXPERIENCE(1)	-.661	.291	5.146	1	.023	.516	.292	.914
	SUBSPECIALTY(1)	.027	.264	.010	1	.920	1.027	.612	1.724
	LOCATION(1)	-.015	.265	.003	1	.954	.985	.586	1.655
	PARENTSHIP(1)	.468	.464	1.016	1	.314	1.597	.643	3.966

a. Variable(s) entered on step 1: SEX, EXPERIENCE, SUBSPECIALTY, LOCATION, PARENTSHIP.

DEPENDENT VARIABLE: TOMATO

INDEPENDENT VARIABLE: SEX, EXPERIENCE, SUBSPECIALTY, LOCATION, PARENTSHIP

Variables in the Equation

		B	S.E.	Wald	df	Sig.	Exp(B)	95% C.I. for EXP(B)	
								Lower	Upper
Step 1[a]	SEX(1)	.734	1.197	.376	1	.540	2.084	.200	21.759
	EXPERIENCE(1)	-1.850	1.195	2.399	1	.121	.157	.015	1.634
	SUBSPECIALTY(1)	-.146	.869	.028	1	.866	.864	.157	4.744
	LOCATION(1)	-2.610	1.223	4.555	1	.033	.074	.007	.808
	PARENTSHIP(1)	-19.823	13879.901	.000	1	.999	.000	.000	.

a. Variable(s) entered on step 1: SEX, EXPERIENCE, SUBSPECIALTY, LOCATION, PARENTSHIP.

DEPENDENT VARIABLE: ORANGE

INDEPENDENT VARIABLE: SEX, EXPERIENCE, SUBSPECIALTY, LOCATION, PARENTSHIP

Variables in the Equation

		B	S.E.	Wald	df	Sig.	Exp(B)	95% C.I. for EXP(B)	
								Lower	Upper
Step 1[a]	SEX(1)	-1.361	.769	3.134	1	.077	.256	.057	1.157
	EXPERIENCE(1)	-1.125	.500	5.064	1	.024	.325	.122	.865
	SUBSPECIALTY(1)	-1.698	.399	18.126	1	.000	.183	.084	.400
	LOCATION(1)	-.532	.407	1.715	1	.190	.587	.265	1.303
	PARENTSHIP(1)	.433	.735	.347	1	.556	1.542	.365	6.515

a. Variable(s) entered on step 1: SEX, EXPERIENCE, SUBSPECIALTY, LOCATION, PARENTSHIP.

DEPENDENT VARIABLE: KIWI OR STRAWBERRY

INDEPENDENT VARIABLE: SEX, EXPERIENCE, SUBSPECIALTY, LOCATION, PARENTSHIP

Variables in the Equation

		B	S.E.	Wald	df	Sig.	Exp(B)	95% C.I. for EXP(B)	
								Lower	Upper
Step 1[a]	SEX(1)	-1.870	.755	6.142	1	.013	.154	.035	.676
	EXPERIENCE(1)	-.817	.395	4.274	1	.039	.442	.204	.958
	SUBSPECIALTY(1)	-1.226	.334	13.446	1	.000	.293	.152	.565
	LOCATION(1)	-.021	.334	.004	1	.950	.979	.509	1.885
	PARENTSHIP(1)	.251	.590	.181	1	.670	1.286	.404	4.089

a. Variable(s) entered on step 1: SEX, EXPERIENCE, SUBSPECIALTY, LOCATION, PARENTSHIP.

SUPPLEMENTARY TABLE S6
Multivariate regression Analysis to evaluate the association of different factors with the time and the waiting period for food introduction of various foods in low- and high-risk children

DEPENDENT VARIABLE: WAITING PERIOD IN HEALTHY CHILDREN

INDEPENDENT VARIABLE: SEX, EXPERIENCE, SUBSPECIALTY, LOCATION, PARENTSHIP

Variables in the Equation

		B	S.E.	Wald	df	Sig.	Exp(B)	95% C.I. for EXP(B)	
								Lower	Upper
Step 1[a]	SEX(1)	.641	.309	4.310	1	.038	1.898	1.036	3.475
	EXPERIENCE(1)	-.060	.251	.057	1	.811	.942	.576	1.540
	SUBSPECIALTY(1)	-.224	.235	.906	1	.341	.799	.504	1.268
	LOCATION(1)	-.029	.237	.015	1	.904	.972	.611	1.545
	PARENTSHIP(1)	-.581	.407	2.035	1	.154	.560	.252	1.242

a. Variable(s) entered on step 1: SEX, EXPERIENCE, SUBSPECIALTY, LOCATION, PARENTSHIP.

DEPENDENT VARIABLE: WAITING PERIOD IN ALLERGIC CHILDREN

INDEPENDENT VARIABLE: SEX, EXPERIENCE, SUBSPECIALTY, LOCATION, PARENTSHIP

Variables in the Equation

		B	S.E.	Wald	df	Sig.	Exp(B)	95% C.I. for EXP(B)	
								Lower	Upper
Step 1[a]	SEX(1)	.314	.463	.460	1	.498	1.369	.552	3.391
	EXPERIENCE(1)	.254	.358	.502	1	.479	1.289	.639	2.599
	SUBSPECIALTY(1)	1.777	.336	27.896	1	.000	5.913	3.058	11.435
	LOCATION(1)	1.066	.345	9.571	1	.002	2.904	1.478	5.705
	PARENTSHIP(1)	-.841	.527	2.542	1	.111	.431	.153	1.213

a. Variable(s) entered on step 1: SEX, EXPERIENCE, SUBSPECIALTY, LOCATION, PARENTSHIP.

QUESTIONNAIRE

Personal Information

1. Sex
male ◯
female ◯

2. Year of birth
[year]

3. Year of graduation from pediatric specialty
[year]

4.Years of work as a pediatrician
[number]

5. Do you have a subspecialty of pediatrics? if Yes please name
[freetext]

6. Place of work (name City/Town and Hospital, University, own practice etc)
[freetext]

7. Number of children
[number]

Complementary feeding order

[This section of the online questionnaire displayed a multi-part **ordering/ranking activity** where users could select from the following items and "place" them in the appropriate month (Birth – Month 18)]

STARCHY / WHEAT
Brown rice (including brown rice flour)
Frumenty
Grains from white flour e.g. Pasta, pastries, etc. from white flour
Oat
Oat (gluten free)
Potato
White rice (including white rice flour)
Whole grains (wheat, barley, rye) e.g. Pasta, pastries, etc
**MILK / DAIRY**

Anthotyro/manouri cheese
Ariani
Cottage cheese
Cow's milk yogurt
Cream cheese (Katiki Domokou)
Drained yogurt
Feta cheese
Fresh cow's milk
Fruit yogurt
Goat's milk yogurt
Gouda cheese
Hard type cheese eg kaseri/graviera/kefalotyri
Kefir
Kid's yogurt
Philadelphia cream cheese
Sheep yogurt
Traditional yogurt with skin

FRUIT

Apple
Apricot
Banana
Cherries
Dried apricots
Dried berries
Dried figs
Dried plums
Figs
Fresh berries
Fresh fruit mixture
Fresh orange juice
Grapes
Kiwi
Mantarine
Melon
Orange
Peach/nectarine
Pear
Pomegranate
Raisins
Strawberries
Watermelon

VEGETABLES

Aubergine
Beetroot
Brocolli

Cabbage
Carrot
Cauliflower
Corn
Green leafy vegetables
Pea
Pepper
Spinach
Tomato
Zucchini

MEAT / MEAT PRODUCTS

Chicken
Cold cuts/Ham/Salami
Game meat - birds (thrush, snipe, partridge, etc.)
Game meat - rabbit, hare, boar
Lamb
Liver
Pork
Rabbit
Veal

FISH / SHELLFISH

Anchovy
Calamari
Clams
Cod
Codfish
Octopus
Salmon
Sardines
Sea bream/bass
Shrimps
Sole
Tuna fish (can)

EGG

Egg omelet/egg eye
Egg with lemon
Hard boiled egg (boiling >6min)
Raw egg

LEGUMES

Beans (all types)
Chickpeas
Fava
Lentils

OILS / LIPIDS

Butter

Corn oil/vegetable oils
Margarine
Olive oil
Olives
Whipping cream

NUTS

Almond paste
Almonds
Cashews
Hazelnut
Hazelnut butter
Peanut
Peanut butter
Pistacchio
Sesame
Sunflower/pumpkin seed
Tachini
Walnuts

SUGAR /SWEETENERS / HONEY

Baby biscuits with sugar
Baby biscuits without sugar
Honey
Sugar/sweets with sugar

FOOD SUPPLEMENTS

Iron
Multivitamin
Omega-3
Probiotic
Vitamin A
Vitamin C
Vitamin D

COOKING INSTRUCTIONS

Salt
Spices (eg pepper, cinammon, etc)
Boiled
Frying
Roasted
Family food with salt
Family food without salt
Mashed food
Fork mashed food
Food in pieces
Ready meal in jar

Additional questions

1. What is the interval between the introduction of one food and the next in a *healthy child*?

1-2 days ☐

2-3 days ☐

3-4 days ☐

4-5 days ☐

5-6 days ☐

6-7 days ☐

2. What is the interval between the introduction of one food and the next in an *allergic child*?

1-2 days ☐

2-3 days ☐

3-4 days ☐

4-5 days ☐

5-6 days ☐

6-7 days ☐

3. Please select for which of the following foods you delay the introduction, *if the child is allergic*:

Kiwi Fruit ☐

Egg ☐

Cow's milk ☐

Yoghurt ☐

Grains (gluten products) ☐

Nuts ☐

Orange ☐

Strawberries ☐

Peanuts ☐

Fish ☐

Other ☐

4. What factors make you vary the general food order guideline you described (eg neurodevelopmental factors)?

[freetext]

Comments

[freetext]
